# EULAR Task Force Recommendations on Annual Cardiovascular Risk Assessment for Patients with Rheumatoid Arthritis: An Audit of the Success of Implementation in a Rheumatology Outpatient Clinic

**DOI:** 10.1155/2015/515280

**Published:** 2015-03-01

**Authors:** Eirik Ikdahl, Silvia Rollefstad, Inge C. Olsen, Tore K. Kvien, Inger Johanne Widding Hansen, Dag Magnar Soldal, Glenn Haugeberg, Anne Grete Semb

**Affiliations:** ^1^Preventive Cardio-Rheuma Clinic, Department of Rheumatology, Diakonhjemmet Hospital, P.O. Box 23 Vinderen, 0319 Oslo, Norway; ^2^Department of Rheumatology, Diakonhjemmet Hospital, P.O. Box 23 Vinderen, 0319 Oslo, Norway; ^3^Department of Rheumatology, Hospital of Southern Norway Trust, P.O. Box 416, 4604 Kristiansand, Norway

## Abstract

*Objective*. EULAR recommendations for cardiovascular disease (CVD) risk management include annual CVD risk assessments for patients with rheumatoid arthritis (RA). We evaluated the recording of CVD risk factors (CVD-RF) in a rheumatology outpatient clinic, where EULAR recommendations had been implemented. Further, we compared CVD-RF recordings between a regular rheumatology outpatient clinic (RegROC) and a structured arthritis clinic (AC). *Methods*. In 2012, 1142 RA patients visited the rheumatology outpatient clinic: 612 attended RegROC and 530 attended AC. We conducted a search in the patient journals to ascertain the rate of CVD-RF recording. *Results*. The overall CVD-RF recording rate was 40.1% in the rheumatology outpatient clinic, reflecting a recording rate of 59.1% in the AC and 23.6% in the RegROC. The odds ratios for having CVD-RFs recorded for patients attending AC compared to RegROC were as follows: blood pressure: 12.4, lipids: 5.0-6.0, glucose: 9.1, HbA1c: 6.1, smoking: 1.4, and for having all the CVD-RFs needed to calculate the CVD risk by the systematic coronary risk evaluation (SCORE): 21.0. *Conclusion*. The CVD-RF recording rate was low in a rheumatology outpatient clinic. However, a systematic team-based model was superior compared to a RegROC. Further measures are warranted to improve CVD-RF recording in RA patients.

## 1. Introduction

The mortality gap between patients with rheumatoid arthritis (RA) and the general population is expanding, a process that is primarily driven by cardiovascular disease (CVD) [1]. Although inflammation has been shown to be a key component in the development of CVD in RA patients [2], there is also a high prevalence of traditional CVD risk factors (CVD-RF) in this patient group [3–6]. Indeed, it has been clearly documented in the general population that if CVD-RFs are identified early and treated successfully, many deaths from CVD may be prevented [7]. Such data are however not available for patients with RA.

The 2010 European League Against Rheumatism (EULAR) task force recommendations for CVD risk management [8], and a more recent updated evidence review [9], propose annual CVD risk assessment of RA patients. Age, gender, and smoking status are already a part of the traditional disease monitoring in the rheumatology setting and thus, as stated by the EULAR task force, a complete CVD risk assessment can easily be incorporated into a routine visit to monitor RA by measuring blood pressure (BP) and adding nonfasting lipids to regular laboratory tests. However, implementation of evidence-based CVD prevention recommendations into clinical practice can be challenging [10, 11] and evidence-practice gaps are persisting [12].

Recording of CVD-RFs is a cornerstone in CVD risk assessments. Therefore, our aim was to evaluate the rate of CVD-RF recording in a rheumatology outpatient clinic that had implemented the recommendations on annual CVD risk assessment for RA patients. Furthermore, we wanted to compare the rate of CVD-RF recording in an arthritis clinic (AC), which is a novel clinical model where CVD-RF recording was performed in a structured, interdisciplinary manner versus a regular rheumatology outpatient clinic (RegROC) that did not include a specific approach to CVD-RF recording.

## 2. Patients and Methods

### 2.1. Patient Population

In 2012, 1142 patients diagnosed with RA fulfilling the ACR/EULAR 2010 criteria [13], visited the rheumatology outpatient clinic of the Hospital of Southern Norway. This outpatient clinic had established an AC, which was a structured clinical model for rheumatic joint disease monitoring. In the AC, work flow was divided into a treatment line with clearly defined work tasks for all health personnel (medical secretaries, nurses, and physicians) involved in the rheumatology consultation. There were no specific inclusion criteria into the AC, and allocation to this clinical model was based solely on the treating outpatient clinic rheumatologist's subjective evaluation of a patient's rheumatic disease. Due to capacity restrictions, only about half of the patients visiting this rheumatology outpatient clinic could be allocated to the AC. Patients who were not invited to the AC attended RegROC consultations.

### 2.2. Recording of CVD-RFs

In 2011, the rheumatology outpatient clinic of the Hospital of Southern Norway implemented the 2010 EULAR recommendations which stated that RA patients should have annual CVD risk assessments. The CVD risk assessment included the recording and evaluation of lipids (total cholesterol (TC), high-density lipoprotein cholesterol (HDL-c), low-density lipoprotein cholesterol (LDL-c), and triglycerides (TG)), fasting glucose, glycated haemoglobin (HbA1c), and blood pressure (BP). Also, comorbidity, smoking status, medication, and history of CVD were recorded by patient self-reporting on computer screens.

In the AC, work tasks related to annual CVD-RF recording were structured as follows: (1) medical secretaries ordered lipid measurements, fasting glucose, and HbA1c as part of routine rheumatology laboratory tests, (2) patient self-reporting on computer screens was performed in the waiting room, prior to the rheumatology consultation, (3) BP measurement was incorporated into the traditional rheumatology nurse consultation, and (4) physicians assessed CVD risk from data available in the patient journal.

The rheumatology outpatient clinic's standard of annual CVD risk assessment was also applied to RA patients attending RegROC consultations, although various work tasks in CVD-RF recording and assessment in this clinical model were not designated to specific personnel.

### 2.3. Evaluation of Recording of CVD-RFs

We conducted a thorough search for CVD-RFs in the patient journals of all the 1142 RA patients, including recordings of BP measurements, cardioprotective medication, CVD comorbidity, and smoking status from any rheumatology consultation conducted in 2012. Regarding laboratory measurements (lipids, fasting blood glucose, and HbA1c), we allowed the measurements to be recorded at any time in the span of ±2 weeks of any rheumatology consultation in 2012.

Subsequently, we divided the 1142 RA patients attending the rheumatology outpatient clinic into two groups, those attending the RegROC and those attending the AC, and compared the rate of CVD-RF recording between the two clinical models.

The systematic coronary risk evaluation (SCORE) is a composite algorithm including age, sex, total cholesterol, systolic BP, and smoking status and provides a 10-year risk estimate for a fatal coronary event [14]. In the absence of national guidelines, EULAR recommends the use of the SCORE algorithm for CVD risk assessment [15]. Accordingly, we considered the patient journal to have a complete CVD-RF profile when the variables included in the SCORE algorithm were recorded.

Finally, to evaluate if an allocation bias to either clinic based on the patients' CVD risk profile existed, we compared the levels of the various CVD-RFs in patients attending the AC versus patients attending RegROC.

### 2.4. Statistics

The data are presented as crude data and the results are expressed as mean ± SD and median (IQR) for normally and non-normally distributed characteristics, respectively. Mann-Whitney U test was used for comparison of the data. The odds ratio [OR with 95% confidence interval (95% CI)] for the CVD-RF being recorded was calculated by logistic regression adjusting for age and gender. Data analyses were performed using IBM SPSS version 20.

## 3. Results

### 3.1. Rate of CVD-RF Recording

**Figure 1:**
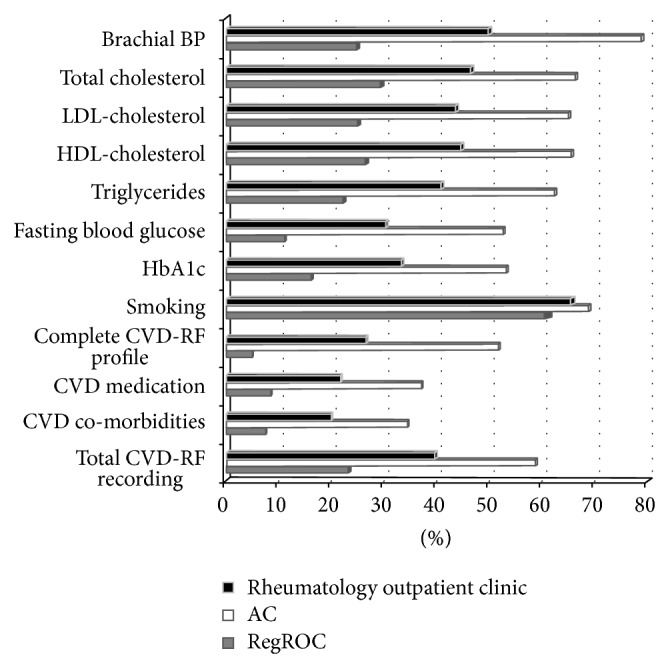
Recording rate of cardiovascular risk factors, medication, and co-morbidity. ROC: rheumatology outpatient clinic of the Hospital of Southern Norway, RegROC: regular rheumatology outpatient clinic, AC: arthritis clinic, BP: blood pressure, LDL: low-density lipoprotein, HDL: high-density lipoprotein, HbA1c: glycated haemoglobin, CVD-RF: Cardiovascular disease risk factor, complete CVD-RF profile: age, sex, total cholesterol levels, smoking status and systolic blood pressure, CVD: cardiovascular disease, CVD medication: antihypertensives and statins, CVD co-morbidities: hypertension, angina pectoris, acute myocardial infarction, percutaneous coronary intervention, coronary artery bypass graft surgery, cerebrovascular accident, and premature familiar cardiovascular disease.

The evaluation of CVD-RF recording in the rheumatology outpatient clinic, as well as in the two clinical models, AC and RegROC, is presented in Table 1.  Figure 1 shows the recording rate for the various CVD-RFs. For the 1142 RA patients attending the rheumatology outpatient clinic at the Hospital of Southern Norway in 2012, the total rate of recording of CVD-RFs was 40.1% and only 26.9% (*n* = 307) of the patients had a complete CVD-RF profile. More specifically, the recording rates for the various CVD-RFs were BP: 50.4%, the various lipids: 41.3–47.0%, fasting blood glucose: 30.7%, HbA1c: 33.7%, smoking status: 66.2%, cardioprotective medication: 22.0%, and CVD comorbidities: 20.2%.

For patients attending the AC, the total CVD-RF recording rate was 59.1%, and the corresponding rate for patients attending RegROC consultations was 23.6%. The odds ratio (OR) for the recording of specific CVD-RFs in patients attending the AC (*n* = 530) versus patients attending RegROC was consistently significant for all CVD-RFs (Table 1). Finally, the OR for having a complete CVD-RF profile was 21.0 (95% CI = 14.0; 31.3).

### 3.2. Level of Recorded CVD-RFs

When comparing the levels of CVD-RFs in patients attending consultations in the AC versus those attending the RegROC (Table 2), we found a significant age and gender difference (*P* = 0.05 and *P* = 0.002, resp.). However, no significant differences concerning BP, lipids, fasting blood glucose, HbA1c, use of cardioprotective medication, presence of CVD comorbidity, the Modified Health Assessment Questionnaire (MHAQ) score, disease duration, or the estimated risk of future coronary events (calculated by SCORE) were revealed between patients attending consultations in the AC and RegROC.

## 4. Discussion

The implementation of effective CVD-RF recording and CVD risk assessment in daily clinical rheumatological practice is an important first step in the process of augmenting the prevention of CVD in RA patients. We have shown that the overall CVD-RF recording was poor in a rheumatology outpatient clinic despite having a standard of annual CVD risk assessment in line with EULAR recommendations. However, the effectiveness of CVD-RF recording was enhanced when patients attended a structured clinical model with clearly defined work tasks, such as the AC.

Nevertheless, even in the AC, the CVD-RF recording was far from complete. In a study from 9 European countries, Ludt et al. [16] reported an impressive rate of CVD-RF recording in 3723 high CVD risk patients from the general (non-RA) population; 92.5% had BP, 83.9% had cholesterol, 75.5% had glucose, and 77.3% had smoking status recorded in their patient journals. When comparing our results to these findings, we conclude that the implementation of systematic, team-based model for RA patients, and CVD-RF recording remained suboptimal. Accordingly, we argue that further measures are necessary to optimize the rate of CVD-RF recording in rheumatology outpatient clinics. In this regard, orchestrated efforts to implement guidelines and recommendations have been shown to be more effective than single strategies [17]. Educating health personnel may be an important measure in such implementation schemes [17, 18]. Indeed, it has been shown that education meetings (conferences, workshops, seminars, symposia, and courses for health professionals) and educational outreach visits by trained persons to health professionals can increase the uptake of recommended care by as much as 10% and 21%, respectively [12, 19]. We advocate that future attempts to implement CVD-RF recording and CVD risk assessment should be undertaken as a part of orchestrated efforts including campaigns and lectures/meetings on CVD risk and CVD-RF for rheumatic health personnel.

Rheumatology nurses played an important role in CVD-RF recording in the AC. The role of nurse-based consultations in rheumatology outpatient clinics has increasingly been acknowledged as they have been shown to bring added value to patients' outcomes at a lower price [20, 21]. Indeed, a recently published study by Primdahl et al. showed that CVD-RF recording in RA patients can be achieved in a 30-minute nursing consultation in addition to the patients' normal follow-up visits [6]. Furthermore, in diabetes clinics, nurse consultations focusing on CVD risk are common and beneficial effects on CVD-RF levels have been reported [22, 23]. Unfortunately, providing all RA patients with an additional 30-minutes consultation to screen for CVD-RFs would result in high cost and be time-consuming. On the contrary, incorporating the CVD-RF screening into traditional rheumatology consultations would be more cost-efficient [24]. However, these important observations concerning the role of the nurse in CVD risk management should undoubtedly be taken into consideration when designing optimal and feasible strategies for CVD prevention in RA patients.

Smoking status, a disease variable that has traditionally been a part of clinical rheumatological practice, was the only CVD-RF that was nearly as effectively recorded in the RegROC as in the AC. This finding underlines the importance of a structured approach to the implementation of disease variables and work processes that are not part of the traditional rheumatology practice, in this case CVD-RF recording.

Considering the low recording rate of cardioprotective medication and presence of CVD comorbidity, we may not have the statistical power to fully exclude the possibility that the patients in the AC had a higher CVD burden that lead to an increased focus on CVD-RF recording in these patients. However, as the AC was primarily a clinical model for rheumatologic disease evaluation, we argue that such allocation bias is not likely. Moreover, as we did not have information concerning the time spent to assess and manage patients in the two clinical models, there is a potential that there was more time per patient in the AC than in the RegROC, which may have improved the feasibility of CVD-RF recording in the AC. A further potential limitation to our study lies in that it did not include rheumatology disease activity measures. Nevertheless, as we found no significant differences in MHAQ or disease duration between AC and RegROC, we can presumably rule out the possibility that the differences in CVD-RF recording rates in the AC and RegROC were biased by more frequent CVD-RF recording in RA patients with higher disease related disability and longer disease duration.

This audit provides an insight into the success rate of implementation of guidelines and recommendations on CVD risk management into the speciality of rheumatology. Furthermore, we have highlighted the important elements that may optimize the implementation of such schemes. However, as this audit reflects what occurs in one institution in Norway, one might raise questions concerning the generalizability of our results. More elaborate studies and projects are therefore warranted to further uncover the optimal approach to implementation of CVD risk management into the field of rheumatology.

## 5. Conclusion

We conclude that the overall CVD-RF recordings were low in a rheumatology outpatient clinic. Despite the increased rate of CVD-RF recording in a structured team-based model compared to a regular clinic; it was still suboptimal. There is a huge unmet need for systems improving CVD-RF recording, which is the first step in the management of the high CVD risk in patients with RA.

## Figures and Tables

**Table 1:** Cardiovascular risk factors recorded in patients attending a rheumatology outpatient clinic.

Risk factor recorded in the patient journal *n* (%)	ROC (*n* = 1142)	AC (*n* = 530)	RegROC (*n* = 612)	OR^*^ (95% CI)	AC versus RegROC *P* value^*^
Blood pressure	576 (50.4)	421 (79.4)	155 (25.3)	12.36 (9.27, 16.48)	<0.001
Total cholesterol	537 (47.0)	354 (66.8)	183 (29.9)	5.02 (3.89, 6.48)	<0.001
LDL-cholesterol	503 (44.1)	347 (65.5)	156 (25.5)	5.87 (4.53, 7.62)	<0.001
HDL-cholesterol	515 (45.1)	350 (66.0)	165 (27.0)	5.59 (4.31, 7.23)	<0.001
Triglycerides	472 (41.3)	333 (62.8)	139 (22.7)	6.02 (4.63, 7.82)	<0.001
Fasting blood glucose	351 (30.7)	281 (53.0)	70 (11.4)	9.11 (6.71, 12.35)	<0.001
HbA1c	385 (33.7)	284 (53.6)	101 (16.5)	6.10 (4.62, 8.04)	<0.001
Smoking	756 (66.2)	378 (69.3)	378 (61.8)	1.44 (1.12, 1.85)	0.05
Complete risk profile included in the SCORE algorithm	307 (26.9)	276 (52.1)	31 (5.1)	20.97 (14.04, 31.33)	<0.001
Cardioprotective medication	251 (22.0)	198 (37.4)	53 (8.7)	6.31 (4.52, 8.82)	<0.001
CVD comorbidities	231 (20.2)	184 (34.7)	47 (7.7)	6.43 (4.53, 9.14)	<0.001

^*^Adjusted for age and gender.

ROC: rheumatology outpatient clinic of the Hospital of Southern Norway, RegROC: regular rheumatology outpatient clinic, AC: arthritis clinic, OR: odds ratio, CI: confidence interval, CVD: Cardiovascular disease, LDL: low-density lipoprotein, HDL: high-density lipoprotein, HbA1c: glycated haemoglobin, complete risk profile: complete lipid values (total cholesterol, HDL-cholesterol, LDL-cholesterol, and triglycerides), smoking, and blood pressure, SCORE: systematic coronary risk evaluation, cardioprotective medication: antihypertensives and statins, and CVD comorbidities: hypertension, angina pectoris, acute myocardial infarction, percutaneous coronary intervention, coronary artery bypass graft surgery, cerebrovascular accident, and premature familiar cardiovascular disease.

**Table 2:** Traditional CVD risk factors, medication, and CVD comorbidities.

	AC group (*n* = 530)	RegROC (*n* = 612)	*P* value
Sex (male) *n* (%)	151 (29.2)	208 (34.3)	0.05
Age years (median, IQR)	62.0 (53.0–70.0)	66.0 (52.8–70.0)	0.002
Disease duration in years (median, IQR)	7.0 (3.0–17.0)	7.0 (3.0–15.0)	0.10
MHAQ (mean ± SD)	0.47 ± 0.47	0.51 ± 0.53	0.13
CVD risk factors (mean ± SD)			
Total cholesterol (mmol/L)	5.55 ± 1.19	5.42 ± 1.20	0.24
LDL-c (mmol/L)	3.32 ± 1.03	3.21 ± 1.00	0.27
HDL-c (mmol/L)	1.66 ± 0.50	1.69 ± 0.51	0.64
Triglycerides (mmol/L)	1.40 ± 0.74	1.49 ± 0.87	0.32
Systolic BP (mmHg)	137.2 ± 19.3	137.1 ± 20.1	0.96
Diastolic BP (mmHg)	82.1 ± 9.3	82.7 ± 11.1	0.49
Fasting glucose (mmol/L)	5.71 ± 1.64	5.75 ± 1.60	0.85
HbA1c (%)	5.71 ± 0.85	5.74 ± 0.83	0.79
Smoking *n* (%)^†^	61/367 (16.6)	56/378 (14.8)	0.50
CVD risk assessment (mean ± SD)			
10-year risk calculated by SCORE in %	4.00 ± 4.5	3.71 ± 3.48	0.64
CVD medication/comorbidities: *n*/*N* (%)^†^ *n*: patients using medication/having CVD *N*: patients with medication/CVD recorded			
Statins	54/198 (27.3)	12/53 (22.6)	0.50
Antihypertensives	56/198 (28.3)	14/53 (26.4)	0.79
Hypertension	48/184 (26.1)	10/47 (21.3)	0.50
Angina pectoris	4/184 (2.2)	1/47 (2.1)	0.98
AMI	4/184 (2.2)	0/47 (0)	0.31
PCI/CABG	6/184 (3.3)	1/47 (2.1)	0.69
CVA	5/184 (2.7)	4/47 (8.5)	0.07
Premature familiar CVD	36/184 (19.6)	4/47 (8.5)	0.07

^†^Presented as the fraction and percent in patients who had cardiovascular risk factors recorded.

RegROC: regular rheumatology outpatient clinic, AC: arthritis clinic, MHAQ: Modified Health Assessment Questionnaire, CVD: Cardiovascular disease, AMI: acute myocardial infarction, PCI: percutaneous coronary intervention, CABG: coronary artery bypass graft surgery, CVA: cerebrovascular accident, CVD: cardiovascular disease, LDL-c: low-density lipoprotein cholesterol, HDL-c: high-density lipoprotein cholesterol, BP: blood pressure, HbA1c: glycated haemoglobin, and SCORE: systematic coronary risk evaluation.

## References

[1] Gonzalez A., Kremers H. M., Crowson C. S. (2007). The widening mortality gap between rheumatoid arthritis patients and the general population. *Arthritis & Rheumatism*.

[2] Crowson C. S., Liao K. P., Davis J. M. (2013). Rheumatoid arthritis and cardiovascular disease. *The American Heart Journal*.

[3] Myasoedova E., Crowson C. S., Kremers H. M. (2011). Lipid paradox in rheumatoid arthritis: the impact of serum lipid measures and systemic inflammation on the risk of cardiovascular disease. *Annals of the Rheumatic Diseases*.

[4] Giles J. T., Allison M., Blumenthal R. S. (2010). Abdominal adiposity in rheumatoid arthritis: association with cardiometabolic risk factors and disease characteristics. *Arthritis and Rheumatism*.

[5] Goodson N. J., Farragher T. M., Symmons D. P. M. (2008). Rheumatoid factor, smoking, and disease severity: associations with mortality in rheumatoid arthritis. *The Journal of Rheumatology*.

[6] Primdahl J., Clausen J., Hørslev-Petersen K. (2013). Results from systematic screening for cardiovascular risk in outpatients with rheumatoid arthritis in accordance with the EULAR recommendations. *Annals of the Rheumatic Diseases*.

[7] Pyörälä K., de Backer G., Graham I., Poole-Wilson P., Wood D. (1994). Prevention of coronary heart disease in clinical practice: recommendations of the task force of the European Society of Cardiology, European Atherosclerosis Society and European Society of Hypertension. *Atherosclerosis*.

[8] Peters M. J., Symmons D. P., McCarey D. (2010). EULAR evidence-based recommendations for cardiovascular risk management in patients with rheumatoid arthritis and other forms of inflammatory arthritis. *Annals of the Rheumatic Diseases*.

[9] Martín-Martínez M. A., González-Juanatey C., Castañeda S. (2014). Recommendations for the management of cardiovascular risk in patients with rheumatoid arthritis: scientific evidence and expert opinion. *Seminars in Arthritis and Rheumatism*.

[10] Graham I. M., Stewart M., Hertog M. G. L. (2006). Factors impeding the implementation of cardiovascular prevention guidelines: Findings from a survey conducted by the European Society of Cardiology. *European Journal of Cardiovascular Prevention and Rehabilitation*.

[11] Semb A. G., Rollefstad S., van Riel P., Kitas G. D., Matteson E. L., Gabriel S. E. (2014). Cardiovascular disease assessment in rheumatoid arthritis: a guide to translating knowledge of cardiovascular risk into clinical practice. *Annals of the Rheumatic Diseases*.

[12] Nieuwlaat R., Schwalm J.-D., Khatib R., Yusuf S. (2013). Why are we failing to implement effective therapies in cardiovascular disease?. *European Heart Journal*.

[13] Aletaha D., Neogi T., Silman A. J. (2010). 2010 rheumatoid arthritis classification criteria: an American College of Rheumatology/European League against rheumatism collaborative initiative. *Annals of the Rheumatic Diseases*.

[14] Conroy R. M., Pyörälä K., Fitzgerald A. P. (2003). Estimation of ten-year risk of fatal cardiovascular disease in Europe: the SCORE project. *European Heart Journal*.

[15] Peters M. J., Symmons D. P., McCarey D. (2010). EULAR evidence-based recommendations for cardiovascular risk management in patients with rheumatoid arthritis and other forms of inflammatory arthritis. *Annals of the Rheumatic Diseases*.

[16] Ludt S., Petek D., Laux G. (2012). Recording of risk-factors and lifestyle counselling in patients at high risk for cardiovascular diseases in European primary care. *European Journal of Preventive Cardiology*.

[17] Francke A. L., Smit M. C., de Veer A. J. E., Mistiaen P. (2008). Factors influencing the implementation of clinical guidelines for health care professionals: a systematic meta-review. *BMC Medical Informatics and Decision Making*.

[18] (2002). Preventive cardiology: how can we do better? Proceedings of the 33rd Bethesda conference. Bethesda, Maryland, USA. December 18, 2001. *Journal of the American College of Cardiology*.

[19] Forsetlund L., Bjørndal A., Rashidian A. (2009). Continuing education meetings and workshops: effects on professional practice and health care outcomes. *Cochrane Database of Systematic Reviews*.

[20] Koksvik H. S., Hagen K. B., Rødevand E., Mowinckel P., Kvien T. K., Zangi H. A. (2013). Patient satisfaction with nursing consultations in a rheumatology outpatient clinic: a 21-month randomised controlled trial in patients with inflammatory arthritides. *Annals of the Rheumatic Diseases*.

[21] van Eijk-Hustings Y., van Tubergen A., Boström C. (2012). EULAR recommendations for the role of the nurse in the management of chronic inflammatory arthritis. *Annals of the Rheumatic Diseases*.

[22] Wallymahmed M. E., Morgan C., Gill G. V., MacFarlane I. A. (2011). Nurse-led cardiovascular risk factor intervention leads to improvements in cardiovascular risk targets and glycaemic control in people with Type 1 diabetes when compared with routine diabetes clinic attendance. *Diabetic Medicine*.

[23] Woodward A., Wallymahmed M., Wilding J., Gill G. (2006). Successful cardiovascular risk reduction in Type 2 diabetes by nurse-led care using an open clinical algorithm. *Diabetic Medicine*.

[24] Gossec L., Salejan F., Nataf H. (2013). Challenges of cardiovascular risk assessment in the routine rheumatology outpatient setting: an observational study of 110 rheumatoid arthritis patients. *Arthritis Care & Research*.

